# Surgical, endovascular, and hybrid treatment of deep femoral artery aneurysm: Three case reports

**DOI:** 10.1002/ccr3.7853

**Published:** 2023-08-28

**Authors:** Hiroki Moriuchi, Takuya Maeda, Masaaki Koide, Yoshifumi Kunii, Kazumasa Watanabe

**Affiliations:** ^1^ Department of Cardiovascular Surgery Seirei Hamamatsu General Hospital Hamamatsu Japan

**Keywords:** deep femoral artery aneurysm, hybrid repair

## Abstract

**Key Clinical Message:**

Deep femoral artery aneurysms (DFAA) are extremely rare. We treated four DFAAs with different procedures including surgical, endovascular, and hybrid surgery. The best treatment should be selected for each individual case.

**Abstract:**

We report three cases of deep femoral artery aneurysms treated with different techniques. Case 1: A 69‐year‐old man with a huge deep femoral artery aneurysm underwent open reconstruction using a 6 mm expanded polytetrafluoroethylen graft. Case 2: A 67‐year‐old man presented with bilateral deep femoral artery aneurysms. The right‐sided rupture was treated with hybrid embolization, while the left aneurysm was treated by endovascular stent‐grafts deployment. Case 3: A 87‐year‐old man with a large deep femoral artery aneurysm underwent simply surgical aneurysmectomy. As there are many treatment options for deep femoral artery aneurysms, a comprehensive preoperative assessment is essential, encompassing an evaluation of symptoms, anatomy, and comorbidities.

## Graphical abstract

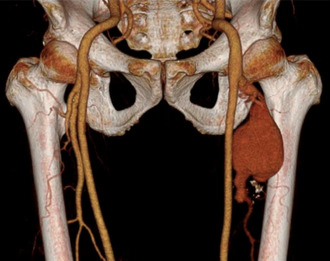

## 1 INTRODUCTION

Degenerative aneurysms of deep femoral arteries are extremely rare, accounting for 1%–2.6% of all femoral artery aneurysms.^1^ Diagnosing them in the early stages is challenging, as they often remain undetected until they reach a substantial size due to their deep location, covered by several muscles.^2^ Deep femoral artery aneurysms (DFAA) can lead to serious complications such as rapid expansion, rupture, and acute lower limb ischemia caused by distal embolism of thrombus. Given the potential for these complications, it is crucial to recommend prompt repair for DFAA.^3^ In this report, we present three cases of DFAA treated either through surgical or endovascular interventions.

## 2 CASE REPORTS

### 2.1 Case 1

**FIGURE 1:**
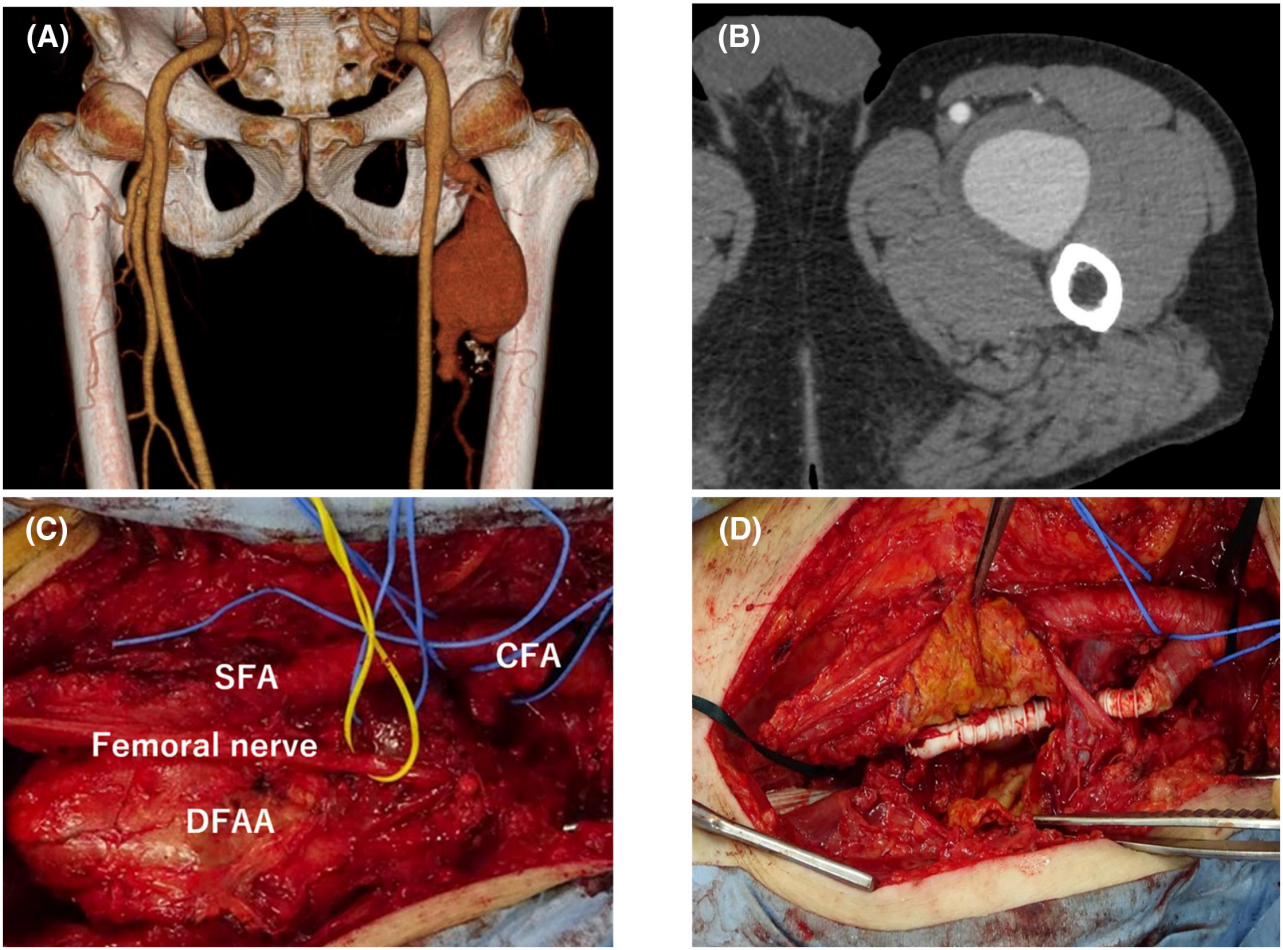
Computed tomography (CT) showed huge left DFAA (A) and abundant thrombus in the sac (B). Femoral nerve was carefully dissected from DFAA (C), and graft replacement was performed (D). DFAA, deep femoral artery aneurysm.

A 69‐year‐old man complained about tenderness in his left thigh and computed tomography angiography (CTA) showed a left large fusiform DFAA (Figure 1A). The aneurysm's maximum size was measured at 60 × 55 mm in diameter and contained a substantial amount of thrombus within the sac (Figure 1B). Given the patient's symptoms and the considerable size of the DFAA, surgical repair was considered essential to prevent rupture and alleviate local compression. The open repair was performed with longitudinal groin incision. The DFAA was found to be adhered to the surrounding tissues, and careful dissection was performed to separate the femoral nerve from the aneurysm (Figure 1C). Additionally, special care was taken to avoid injury to the femoral veins and minimize the risk of perioperative venous thrombosis. The aneurysmectomy was conducted and 6 mm ring‐supported expanded polytetrafluoroethylen (ePTFE) graft was interposed into the DFA (Figure 1D).

### 2.2 Case 2

**FIGURE 2:**
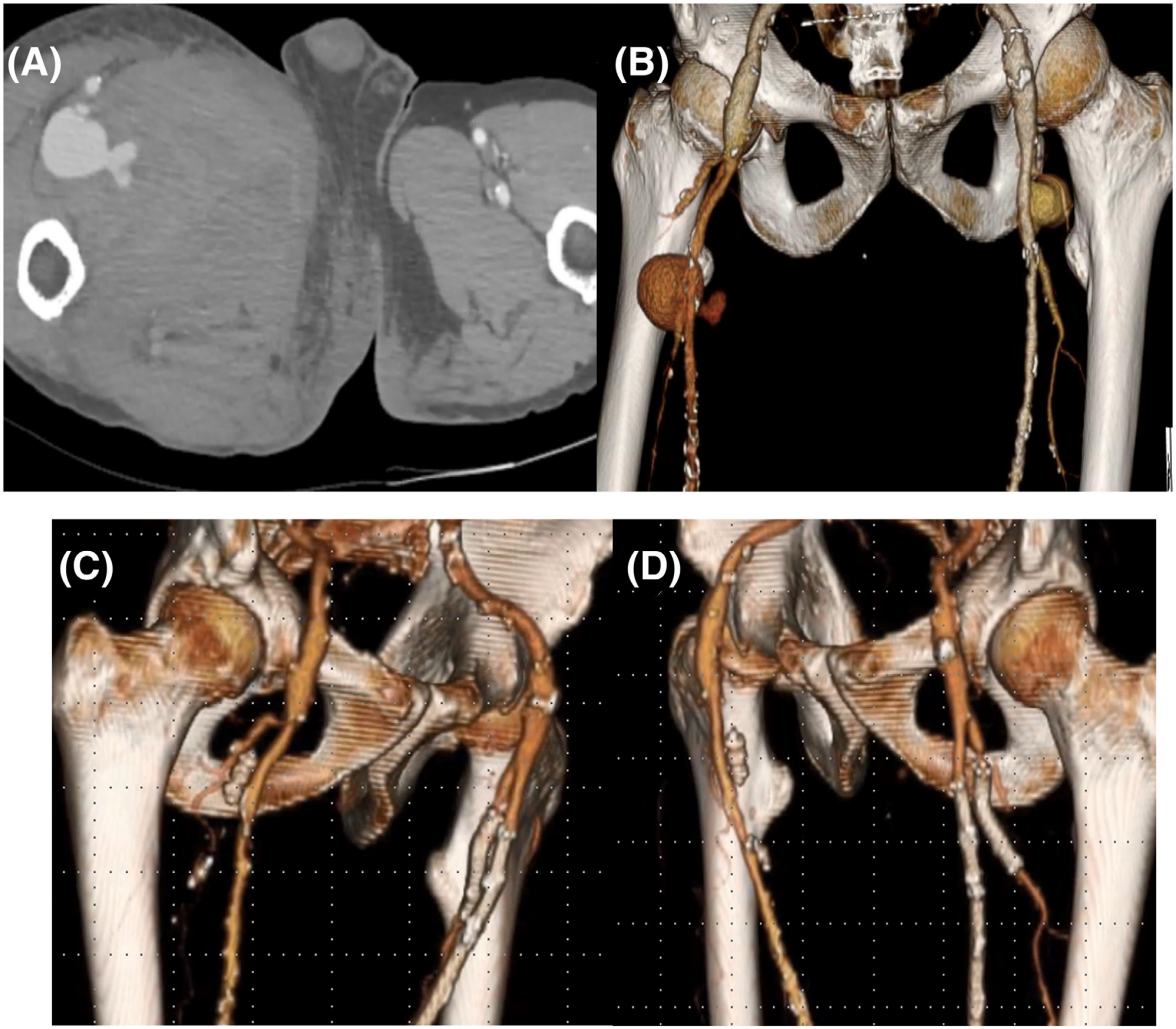
CT revealed bilateral DFAA and ruptured right‐sided aneurysm (A, B). Postoperative CT showed exclusion of DFAA blood flow and patent stent grafts (C, D).

A 67‐year‐old man was referred to our hospital with sudden right thigh pain. CTA revealed bilateral DFAA with the right DFAA found to be ruptured (Figure 2A,B). An Emergency surgery was performed to address the ruptured right DFAA. Right common femoral artery (CFA) and proximal DFA were dissected through a groin incision. A 5Fr. sheath was inserted via the right CFA into the distal artery of DFA. The distal branch of DFAA was embolized with 7 mm Amplutzer vascular plug 4 (Abbott). Finally the proximal DFA was ligated. The patient was discharged with no sign of limb ischemia.

The follow‐up CTA showed the enlargement of the left DFAA measuring 25 mm in diameter and occlusion of left superficial femoral artery (SFA). The left ankle brachial index showed 0.51. To address both conditions simultaneously, endovascular management was chosen as the preferred treatment approach. A 7Fr sheath into the left common femoral artery (CFA) through a small groin incision. First, the occlusive long lesion in the left SFA was treated with balloon angioplasty, followed by the deployment of a 6 × 250 mm stent graft (Viabahn, WL Gore & Associates Inc.). Next the 7Fr sheath was gently advanced to the distal artery of DFAA and a 7 × 500 mm self‐expanding stent graft was deployed from the distal artery to the proximal neck of the DFAA. Postoperative CT revealed exclusion of DFAA blood flow and patent stent grafts (Figure 2C,D). The left ankle brachial index rose to 0.97 and the patient was discharged without any leg ischemic symptoms.

### 2.3 Case 3

**FIGURE 3:**
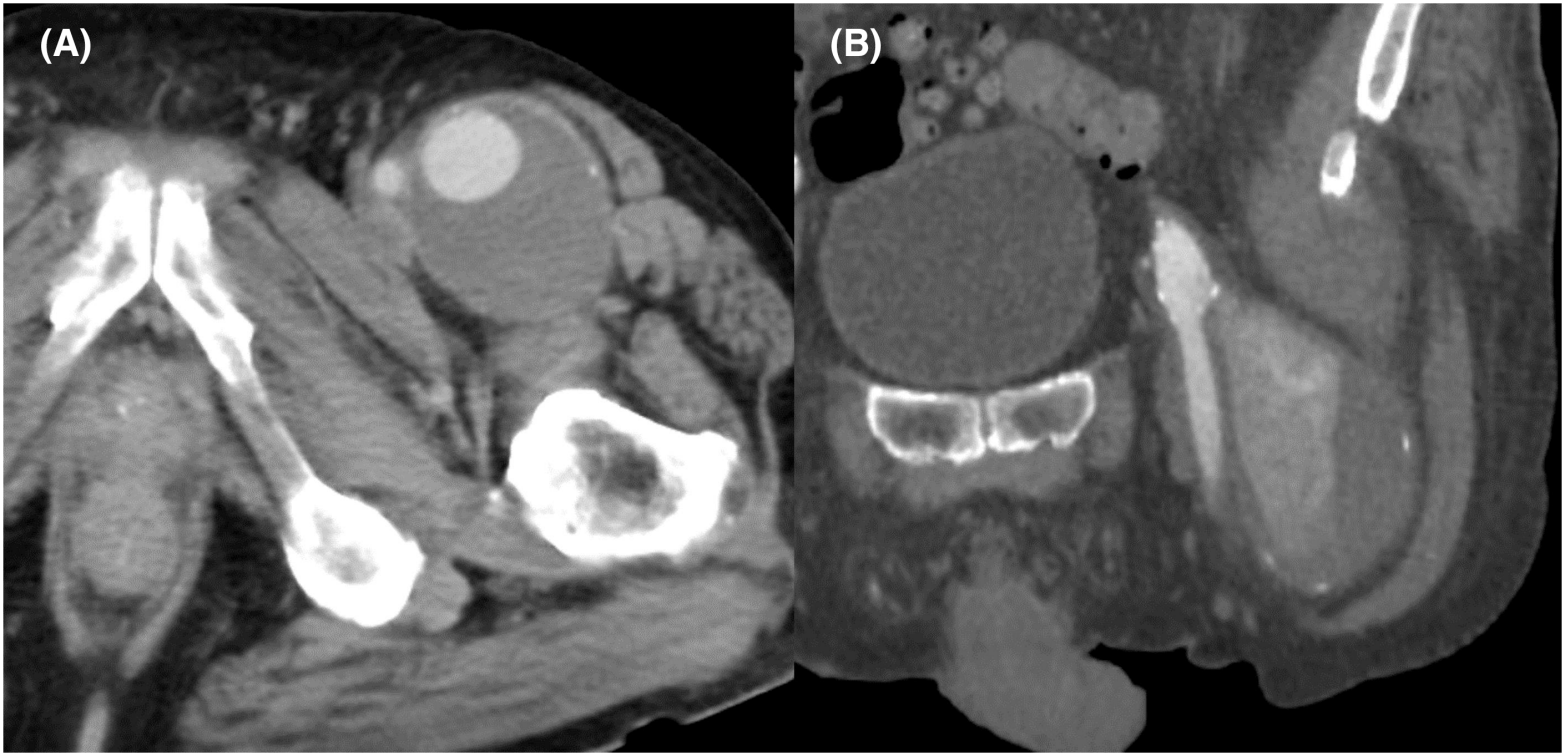
CT showed left DFAA (A) and proximal neck from bifurcation was very short (B).

A 87‐year‐old man was referred to our institute because CTA revealed a left DFAA measuring 50 mm in diameter. Although asymptomatic, the patient had a high clinical frailty scale score of 7, attributed to his advanced age and sequelae from cerebral infarction. Endovascular management was considered to be impossible because the DFAA enlarged proximally at the just bifurcation from the CFA (Figure 3A,B). An aneurysmectomy and proximal and distal ligation of the DFAA were performed. Reconstruction of DFA was not done considering the patient's low activity level and the absence of stenosis in the SFA.

All postoperative courses were uneventful, without lower limb ischemia.

## 3 DISCUSSION

DFAA is rare and difficult to diagnose in the early stage.^1^ DFAA often progresses asymptomatically and is found in large size at presentation because they are located deeply and covered by several muscles.^2^ DFAA can cause complicated conditions such as rapid expansion, rupture, and acute lower limb ischemia due to distal embolism of thrombus, particularly if there is concomitant superficial femoral artery occlusive disease. Reslan and colleagues suggested that repair is always recommended for DFAA because of the possibility of those complications.^3^

Treatment of DFAA is usually open repair consisting of exclusion of aneurysm and reconstruction of DFA with prosthetic graft. The prosthetic grafts are better size matches and patency rates than vein grafts in the femoral artery region.^4^ Preoperative assessment of ipsilateral SFA patency and other regions including iliac artery, popliteal artery, and contralateral side is very important because femoral artery aneurysms are often associated with different aneurysms and bilateral aneurysms.^5^, ^6^

Although aneurysmectomy and graft replacement are preferred, simple ligation or embolization may be reasonable treatment in challenging cases such as ruptured aneurysms, elder patient with poor general condition.^3^ Coil embolization has been reported as useful method if the aneurysm involves distal branches of DFA.^7^, ^8^ However, it is essential to recognize that the patients are at risk for limb ischemia because DFA is an important collateral source to the lower extremity, especially in cases of femoropopliteal artery diseases.^1^

Endovascuolar treatments are attractive for frail patients because of their less invasiveness and using stent‐grafts for DFAA may be effective approach for the preservation of distal perfusion. There are some reports of successful deployment of stent‐grafts to treat DFAA with good short‐term results.^5^, ^6^, ^9^ However, contralateral femoral access or groin incision is often required to deliver stent‐grafts. Further size discrepancy between proximal and distal arteries of DFAA is assumed if aneurysm is large and long, so preoperative assessment using CTA or magnetic resonance angiography is essential. Postoperatively, careful follow‐up of graft patency and local compressive and ischemic symptoms is necessary.

In Case 1, the patient was young with good activity level, and the anatomy of the DFAA was not complicated. Hence, we opted for open repair using an ePTFE graft.

In Case 2, the patient presented with a ruptured right DFAA and exhibited signs of shock. Given the challenging situation of significant bleeding and poor visibility, open repair was deemed technically difficult. Therefore, we opted for endovascular management, wherein we embolized the distal artery of the DFAA using an Amplatzer vascular plug and performed proximal direct ligation. The left DFAA was short length with enough both proximal and distal landing zone and SFA was occluded, we performed endovascular treatment with 6 and 7 mm stent‐graft.

In Case 3, the patient was high age with poor general condition and endovascular treatment was considered to be impossible due to a short landing zone. Consequently, we performed only aneurysmectomy. Reconstruction of DFA was not done because the patient had low activity levels and SFA had no stenosis.

## 4 CONCLUSION

We have successfully reported three cases of DFAA treated using different approaches, including open surgical reconstruction, hybrid embolization, and endovascular reconstruction, as well as simple aneurysmectomy. As there are multiple approaches for treatment of DFAA, preoperative assessment is important including symptoms, anatomy, the presence of different anerurysm, ischemic arterial disease, and comorbidities. Further study is necessary to evaluate long‐term results of treatment of DFAA.

## AUTHOR CONTRIBUTIONS

**Hiroki Moriuchi:** Conceptualization; data curation. **Takuya Maeda:** Conceptualization; writing – review and editing. **Masaaki Koide:** Supervision. **Yoshifumi Kunii:** Supervision. **kazumasa watanabe:** Conceptualization.

## FUNDING INFORMATION

None.

## CONFLICT OF INTEREST STATEMENT

The authors report no conflict of interest.

## CONSENT

Written informed consent was obtained from the patient to publish this report in accordance with the journal's patient consent policy.

## Data Availability

None.
